# Electrical storm due to Epstein-Barr virus-induced lymphoma of a transplanted heart: a case report

**DOI:** 10.1093/ehjcr/ytac212

**Published:** 2022-06-13

**Authors:** Etienne Charbonneau, Vincent Galand, Erwan Flécher

**Affiliations:** Department of cardiothoracic and vascular surgery, Rennes University Hospital, Rennes, France; Department of cardiology, Rennes University Hospital, Rennes, France; Department of cardiology, Rennes University Hospital, Rennes, France; Department of cardiothoracic and vascular surgery, Rennes University Hospital, Rennes, France

**Keywords:** Lymphoma, Heart transplant, Epstein-Barr virus, Electrical storm, Case report

## Abstract

**Background:**

Cancers, and specifically lymphomas, are one of the main causes of morbidity and mortality after heart transplantation. Sixteen percent of heart transplant recipients develop cancer within 5 years and lymphomas represent 10% of these patients.

**Case summary:**

We report the case of an Epstein–Barr Virus–induced primary cardiac lymphoma on the graft. The patient initially present an electrical storm quickly controlled under medical treatment. The multimodal exploration led us to the diagnosis of lymphoma. The lymphoma has spread quickly in spite of the numerous treatments we have tried and has resulted in arrhythmia complications.

**Discussion:**

This case report highlights the challenging management of heart transplant lymphoma and its treatment. Primary cardiac lymphoma on the graft is highly rare with poor prognosis and arrhythmia complications.

**Learning points**

- Isolated primary cardiac lymphoma on the graft is a rare and extremely serious complication of heart transplant.
- The optimal immunosuppressive strategy for heart transplant remains uncertain.

## Introduction

Cancers remain one of the main causes of long-term mortality after heart transplantation. According to the International Society of Heart and Lung Transplantation, 16% of transplant recipients develop cancer within 5 years and almost 30% within 10 years.^1,2^ Among this population, 50% develop cutaneous cancers but lymphomas are frequent as well, representing 10% of these patients.^2^ Interestingly, the incidence of lymphoma does not regularly increase during follow-up but is more important during the first 5 years following transplantation.^3^ Notably, post-cardiac transplant lymphomas are more likely extranodal with lesions of the central nervous system, lungs, or liver.^3^ However, isolated cardiac graft lymphomas are extremely unusual and have been poorly reported so far. We aimed to describe the case of a patient who developed an isolated graft lymphoma after receiving heart transplantation.

## Timeline

**Figure:**
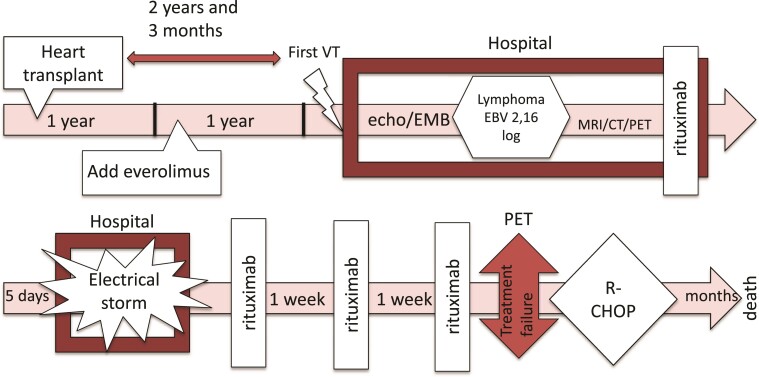


## Case summary

In 2017, a 52-year-old caucasian man without previous medical history was hospitalized for acute advanced heart failure due to massive myocardial infarction. At that time, he was on the list for high emergency heart transplantations but did not receive a graft within the allocated time in France for such a procedure and was consequently implanted with a left ventricular assist device (LVAD) as a bridge to transplant. During the first months of LVAD follow-up, two ischaemic strokes without any sequelae occurred. Taking into account these thromboembolic complications under LVAD support, we successfully transplanted the patient in 2017. Epstein–Barr virus (EBV) serologies were mismatched, the donor was positive for EBV and the patient was negative, Both patients were negative for other serologies such as cytomegalovirus, varicella zoster virus, herpes simplex virus, and toxoplasmosis. The patient had no HLA antibodies before the graft and did not devel any, virtual cross-match was negative and the patient did not present any heart graft rejection during follow-up. He had endomyocardial biopsy at regular intervals during his follow-up to rule out rejection, twice a month during the first 6 months, monthly for the following 6 months, then every 3 months during the second year. We used thymoglobulin 1 mg/kg per day during 4 days as induction therapy, followed by low dose of corticosteroid, mycophenolate mofetil 2 g per day and cyclosporin 250 mg per day as background treatment.

After 1 year, renal failure induced by cyclosporin toxicity appeared with creatinine level at 154 μmol/L. Consequently, everolimus 1,5 mg per day was added to use lower cyclosporine dose and prevent renal failure. We switched to a four-drug immunosuppressive regimen with a decrease in both corticosteroid and cyclosporin doses to 10 mg per day and 175 mg per day, respectively, for a residual of 100 ng/mL. The follow-up was simple with a good quality of life.

**Figure 1:**
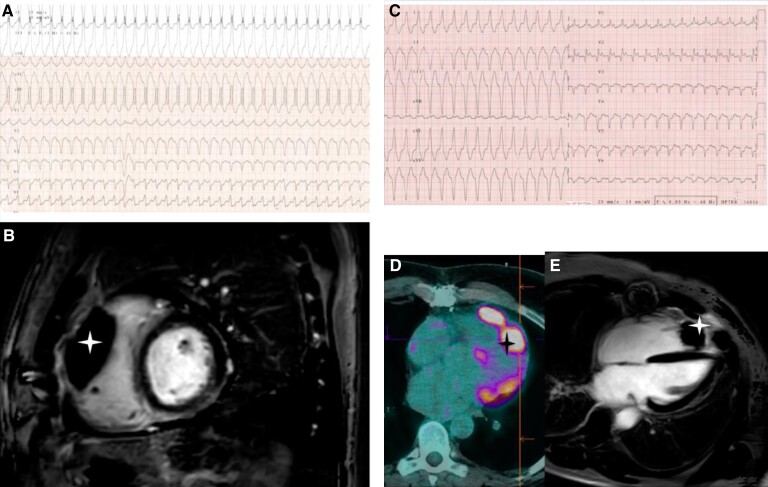
Heart lymphoma imaging and corresponding ventricular tachycardia during electrical storm. (*A*) Ventricular tachycardia from antero-medium node. (*B*) Antero-medium wall node on magnetic resonance imaging (MRI) phase-sensitive T1 inversion recovery (PSIR) sequence small axis plan. (*C*) Ventricular tachycardia from apical node. (*D*) Positron emission tomography 18 fluorodesoxyglucose 4 cavity plan antero-medium and apical nodes. (*E*) Antero-medium and apical nodes on MRI SPIR sequence 4 cavity plan. Stars indicates the VT’s causal nodes.

**Figure 2:**
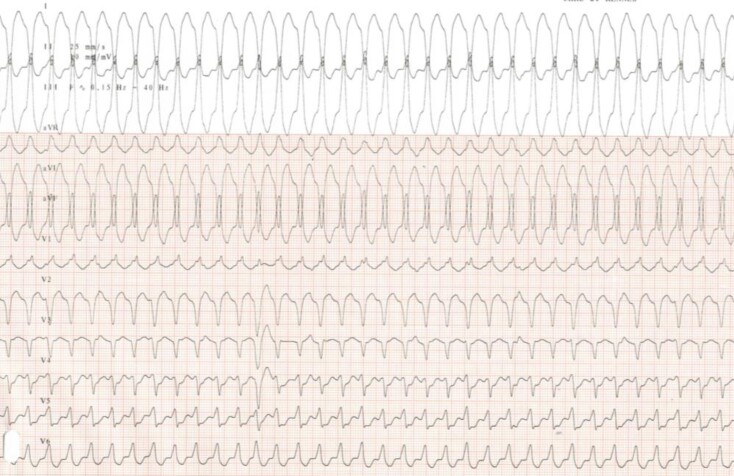
Ventricular tachycardia from antero-medium node.

In December 2019, he was admitted to our hospital for well-tolerated rapid palpitations without any others complaints and especially with no sign of haemodynamic failure or heart failure. He had no signs of heart failure, any peripheral oedema, no jugular venous pulse, no hepatoojugular reflux, and at lung auscultation, we heard normal respiratory sounds. At heart auscultation, we heard a gallop rhythm without other sounds added. We identify ventricular tachycardia (VT) on the electrocardiogram (ECG) (*figure 1A*) (*figure 2*). VT was reduced by a dose of Atenolol. Echocardiographic parameters showed no myocardial dysfunction, no valvular disease, no pericardial effusion, and no significant anomalies. The patient’s baseline ECG was not modified. Further investigations did not demonstrate any abnormalities besides an EBV high replication of 4,16 log copies/mL. He had no electrolytes abnormalities, BNP was normal, but he had a moderate elevation of troponin at 101 pg/mL.

**Figure 3:**
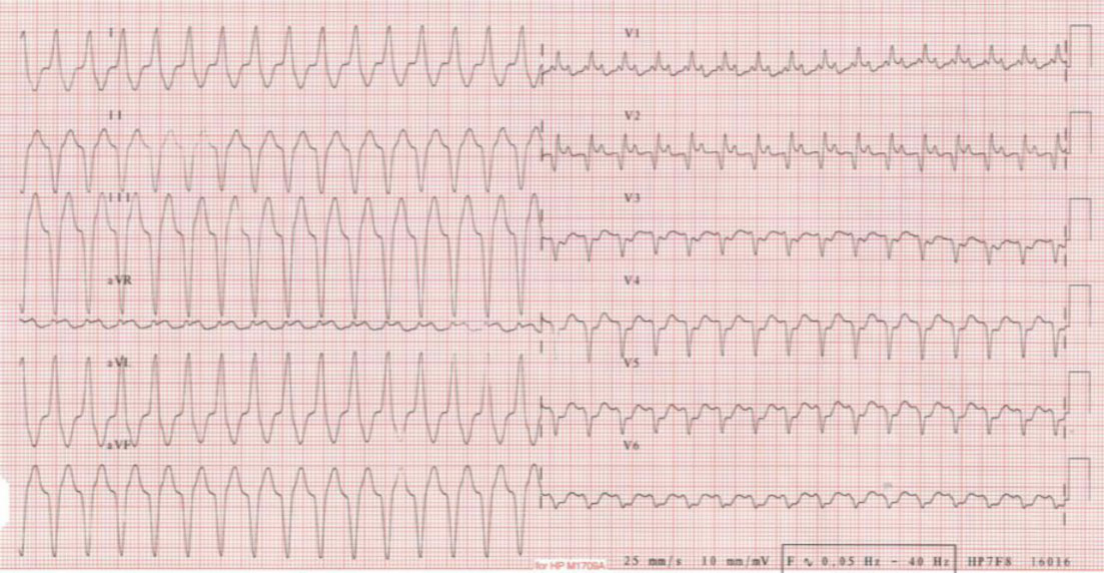
Ventricular tachycardia from apical node.

**Figure 4:**
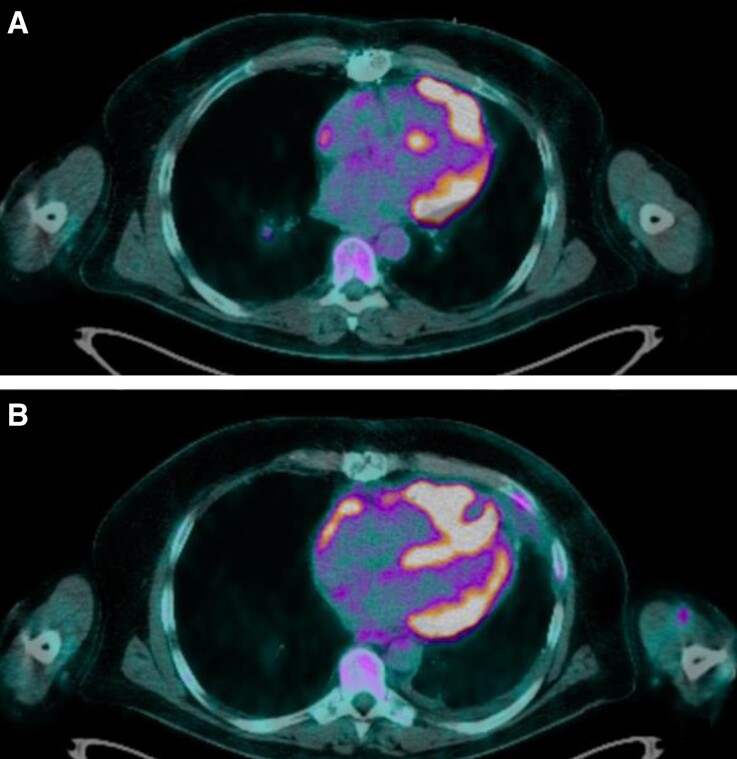
Evolution of lymphoma in 18 fluorodesoxyglucose (FDG) positron emission tomography scan. (*A*) Positron emission tomography 4 cavity plan initial. (*B*) FDG PET-scan 4 cavity plan re-evaluation after treatment with rituximab.

Endomyocardial biopsies were performed quickly and ruled out an acute rejection. Nonetheless, the microscopic appearance revealed a post-transplantation monomorphic lymphoproliferative disorder, EBV-induced, high-grade B cell lymphoma type diffuse large B-cell lymphoma. Coronary angiography was normal. To complete the results of the biopsies, cardiac computed tomography and cardiac magnetic resonance imaging revealed two nodular images developed in the antero-medial and apical wall of the right ventricle and extending intracavitary and in the epicardial area with significant hypermetabolism on the 18FDG-PET (*figure 1*). The heart transplant team met the haematologists in a pluridisciplinary meeting to discuss this complex case and define the therapeutic strategy. First-line therapy was rituximab 375 mg/m^2^ one injection per week for 4 weeks and a reduction of the immunosuppressive drugs to decrease the EBV replication, and 2,95 log copies/mL was the minimal EBV level reached. After the first rituximab injection he returned home, but a few days later, despite optimal medical therapy the patient presented an electrical storm, which was badly tolerated with hypotension, peripheral cyanosis, decreased urine output. Because off haemodynamic instability, he was admitted in the intensive care unit. The first VT which seemed to originiate from the apical node unlike the first episode of arrhythmia was reduced by electrical cardioversion (*figure 1C*) (*figure 3*). Post-shock rhythm was sinus rythm but quickly ventricular arrhythmias recurred, requiring Esmolol infusion. This allowed reduction of VT. After 2 days, Nadolol was introduced with a good tolerance. The patient was discharged from the hospital and finished the first therapeutic line in an outpatient hospitalization unit every week. We then fitted the patient with a life vest, but he quickly stopped wearing it due to the bone pain induced by the chemotherapy. At the end of the treatment a PET re-evaluation (*figure 4*) was performed showing the persistence of an intense hypermetabolism of the three pericardial tumours. Second therapeutic line was R-CHOP protocol (rituximab 375 mg/m^2^, doxorubicin 50 mg/m^2^, cyclophosphamide 750 mg/m^2^, and vincristine 1,4 mg/m^2^) every 21 days. Successive chemotherapy cycles were ineffective with a tachyphylaxis situation and a quick progression of the tumoural lesions assessed by cardiac imaging and significant EBV replication of 5,4 log copies/mL. The patient died from a sudden death at home 3 months after the diagnosis and only after two cycles of R-CHOP.

## Discussion

Isolated primary cardiac lymphoma is an exceptional post-transplant complication with only very rare cases previously reported to the best of our knowledge.^4^

The estimated prevalence of primary cardiac tumours is 1:2000 autopsies and for secondary tumours 1:100 autopsies.^5^ Primary cardiac lymphoma is a rare malignant disease. Lymphoma composes just 1.3% of all cardiac tumours. The most common histological type of PCL is large B cell lymphoma.^6^ Ventricular arrhythmias are a classic manifestation of cardiac tumours and therefore cardiac lymphomas. A case of rhythmic storm revealing a lymphoma with cardiac extension was described^7^ but no cases of primary cardiac lymphoma. In this case, the echographic diagnosis was difficult, as we initially had no echographic abnormalities. In some cases, echography is described as a good screening tool for lymphoma which appears as a myocardial mass without any specific abdnormality.^8^ However, CMR can help diagnose cardiac lymphoma, typically CMR shows homogeneous tumour enhancement, lymphomas diffusely involved in the right atrioventricular groove and completely surrounding the right coronary artery. Pericardial effusion is common.^9^

The appropriate treatment strategy remains challenging. We decided not to perform a second heart transplantation despite the patient's age, his good general condition and localized tumour with no extracardiac extension. Indeed, only a few publications reported this therapeutic option for primary cardiac tumours but with heterogeneous results.^10,11^ Surgically, a second transplant was at high risk due to immunosuppression, chemotherapy, redo patient. Heart retransplantation requires high doses of immunosuppressive drugs which increases the risk of EBV replication. We considered a second transplantation to be unsafe with an high risk of recurrence on the graft.

We had used rituximab initially. This treatment has many benefits such as short duration of treatment, administration on an outpatient basis, no drug interaction with anti rejection drugs. One of the classically described side effect of rituximab is increased rhythmic risk, but we decided the risk was low compared with the benefit of this treatment.

In second line, we used R-CHOP protocol as recommended.^12^

The patient presented rather many side effects of the R-CHOP therapy such as thrombopenia and bone pain. As the surgical resection is not efficient in the case of cardiac lymphoma we did not consider it.^13^

Considering the immunosuppressive strategy, we chose an immunosuppressive regimen with four drugs (Prednisolone, mycophenolate mofetil, everolimus, cyclosporine) before the lymphoma, to use lower calcineurin inhibitors dose and prevent renal failure.^14^ We added everolimus after 1 year but it could have been added earlier. The everolimus addition should have allowed us to decrease even further the dose of calcineurin inhibitors with lower residual targets than usual and therefore also prevent EBV replication.

Earlier and more severe immunosuppression minimization with addition of everolimus could have beneficial effects.

Rhythmically, the first VT rhythm control was provided by atenolol.

During electrical storm, we used esmolol to reduce TV. After this event, we switched atenolol for a low dose of Nadolol. We did not use cordarone because of the risk of increased cyclosporin renal toxicity in a patient who already presented an altered renal function. The use of beta-blockers was cautious in this patient for whom the adaptation of the heart rate was not physiological. Radiofrequency catheter ablation could be an interesting option for such patients but the procedure was associated with high risks including tumour spreading or ventricular hyperexcitability.^15^ In addition, the penetration of the radiofrequency current would probably be insufficient due to the large tumoral masses. A few teams developed non-invasive VT ablation methods through external radiation. This method could have been interesting in this case as it presents the double benefit of enhanced security of the procedure and of transmural ablation that might have been beneficial to the lymphoma treatment.

## Conclusions

Primary cardiac lymphoma of the graft is a very rare complication with a very poor prognosis. Its management and treatment remain uncertain in this population. Adaptation of immunosuppression and development of non-invasive radiofrequency ablation techniques might be interesting research tracks in the future.

## Lead author biography

**Figure:**
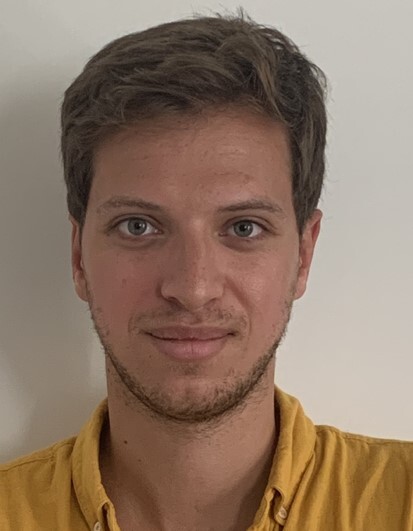


I’m cardiology resident at CHU of Rennes in France, specialized in cardiovascular imaging and critical care in heart failure. My research work focuses on LVAD and heart transplant. I have successively carried out internships in specialized services in the field off pulmonary arterial hypertension, heart transplant, intensive care unit, and mechanical circulatory support devices.

## Supplementary Material

ytac212_Supplementary_Data

## Notes

**Slide sets:** A fully edited slide set detailing these cases and suitable for local presentation is available online as Supplementary data.

**Consent:** The authors have obtained written consent from the patient in accordance with COPE guidelines.

**Funding:** None declared.
