# Supplementary material for: Electrical storm due to Epstein-Barr virus-induced lymphoma of a transplanted heart: a case report
Source: Eur Heart J Case Rep. 2022 Jun 13;6(6):ytac212. doi: 10.1093/ehjcr/ytac212 (PMC9210942; doi:10.1093/ehjcr/ytac212)
Supplement: ytac212_Supplementary_Data [file ytac212_supplementary_data.pptx]

## Slide 1
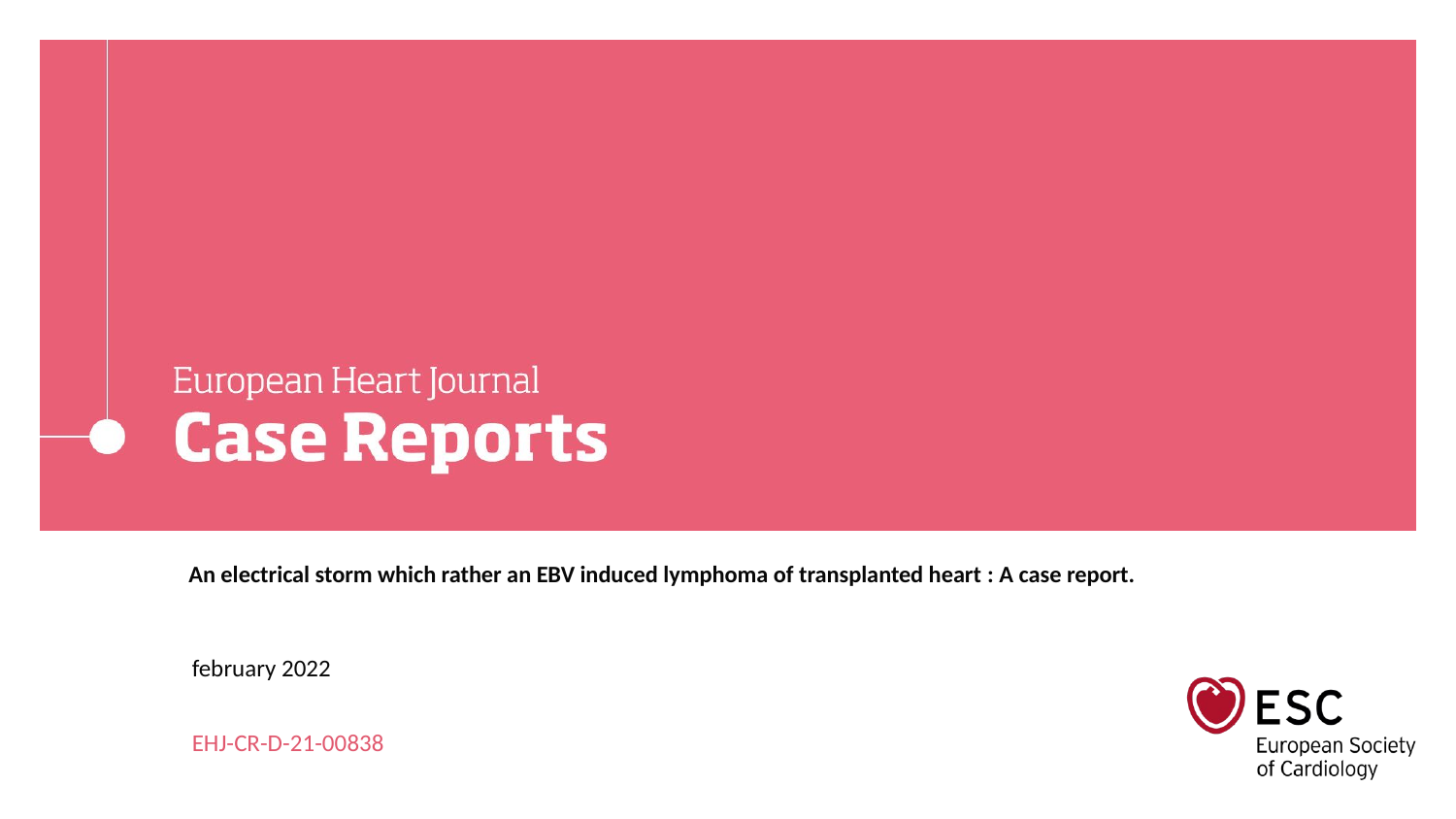

# An electrical storm which rather an EBV induced lymphoma of transplanted heart : A case report.
february 2022
EHJ-CR-D-21-00838

## Slide 2
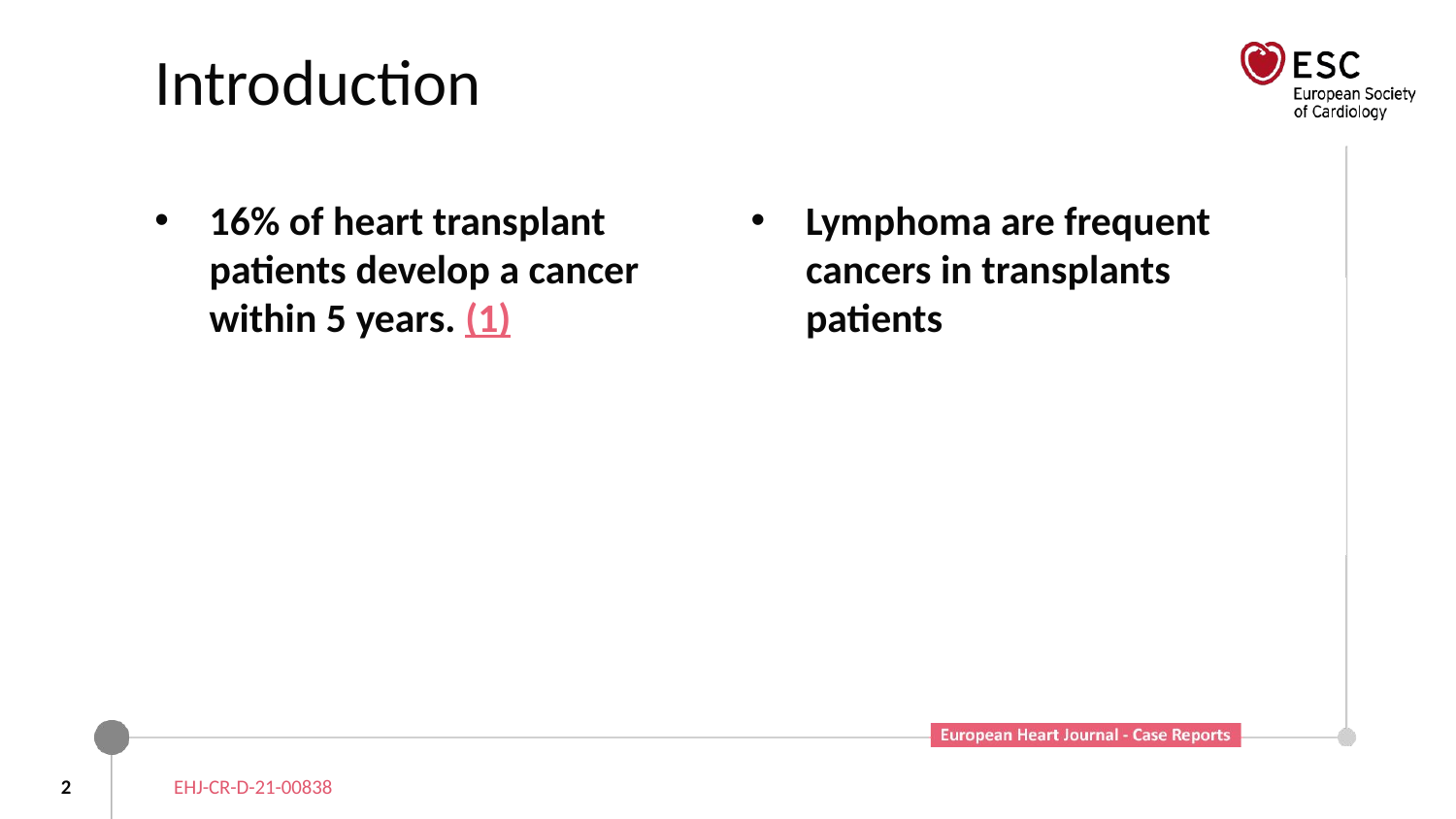

# Introduction
16% of heart transplant patients develop a cancer within 5 years. (1)
Lymphoma are frequent cancers in transplants patients
2
 EHJ-CR-D-21-00838

## Slide 3
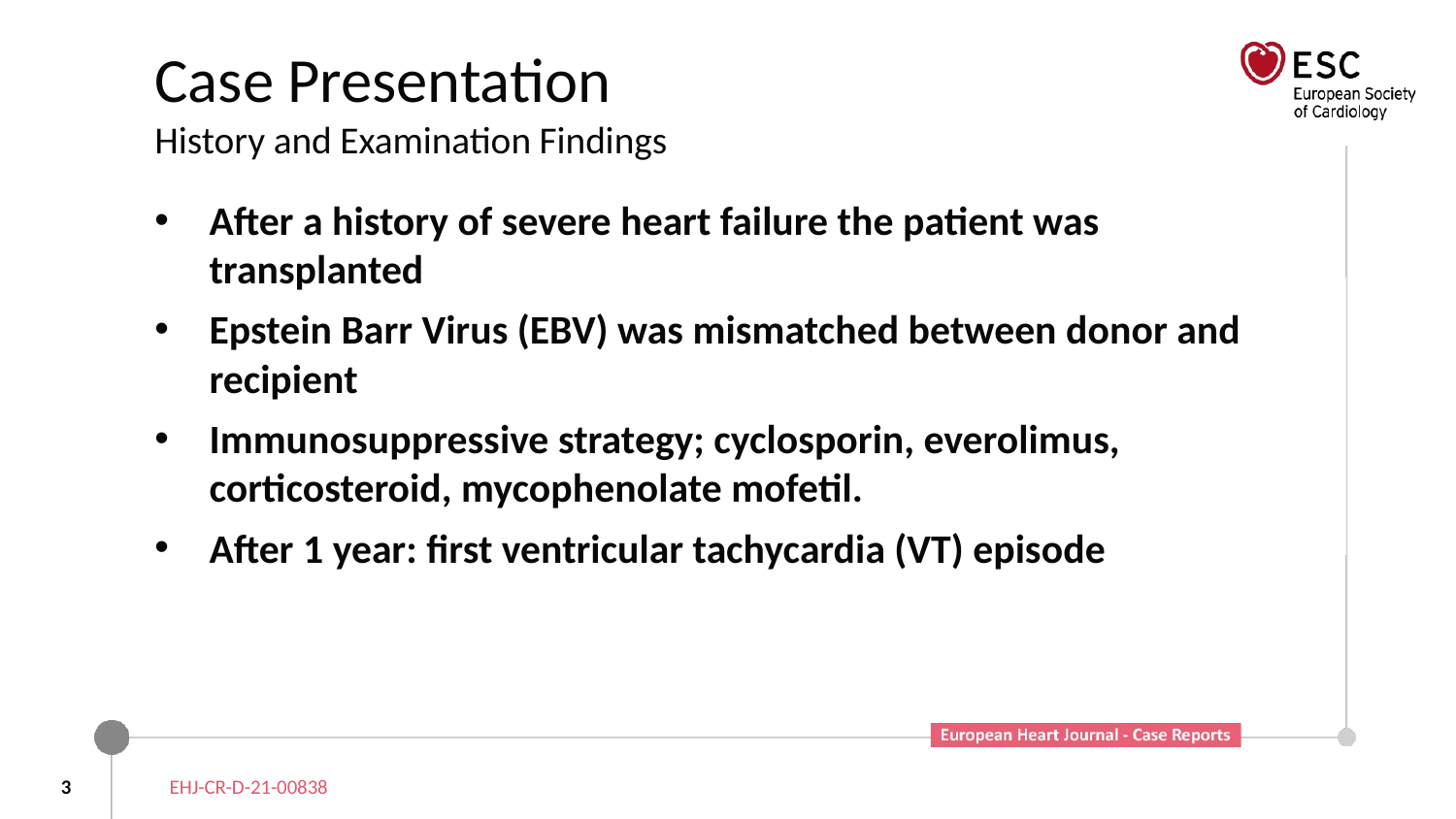

# Case Presentation History and Examination Findings
After a history of severe heart failure the patient was transplanted
Epstein Barr Virus (EBV) was mismatched between donor and recipient
Immunosuppressive strategy; cyclosporin, everolimus, corticosteroid, mycophenolate mofetil.
After 1 year: first ventricular tachycardia (VT) episode
3
EHJ-CR-D-21-00838

## Slide 4
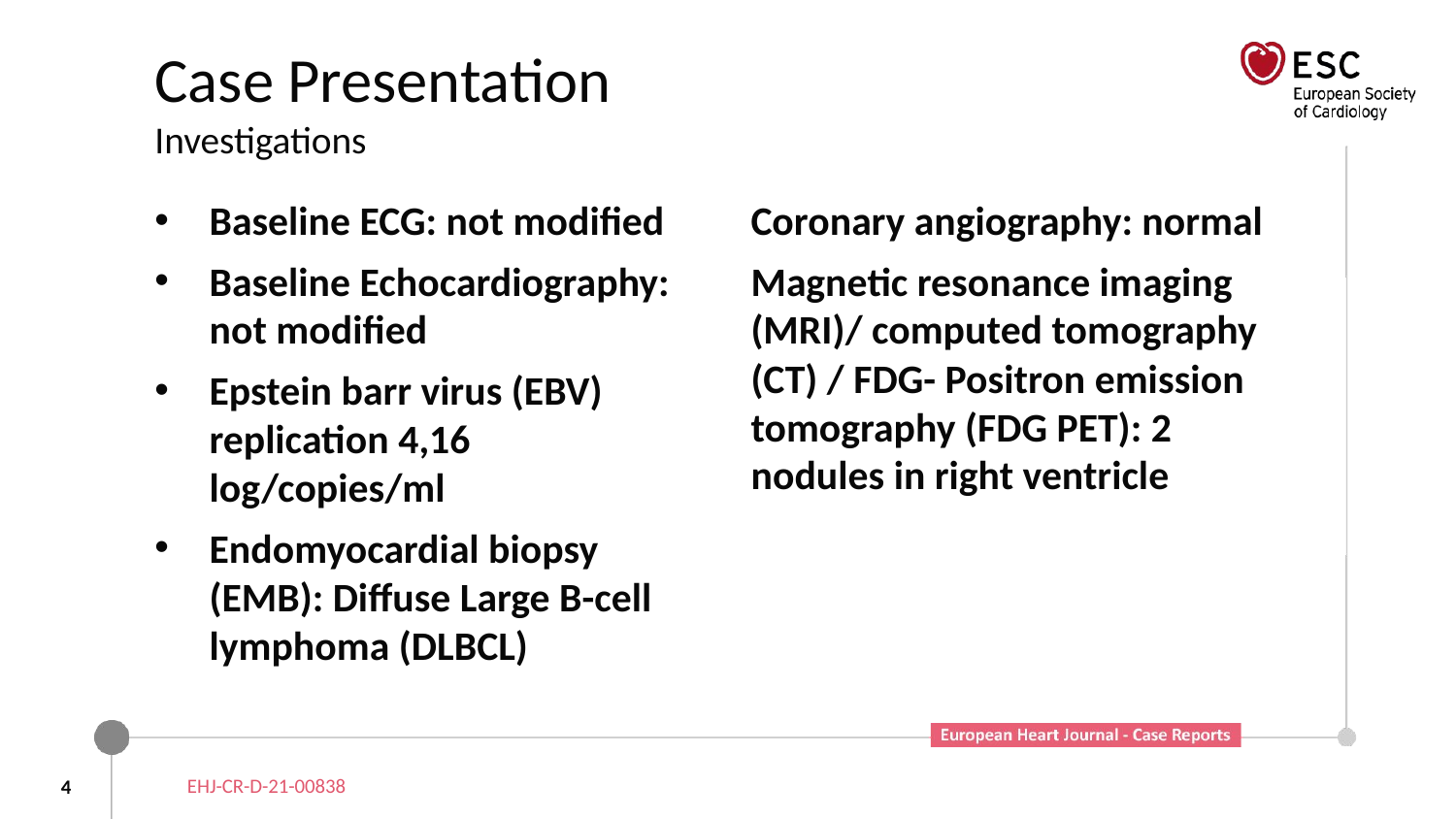

# Case Presentation Investigations
Baseline ECG: not modified
Baseline Echocardiography: not modified
Epstein barr virus (EBV) replication 4,16 log/copies/ml
Endomyocardial biopsy (EMB): Diffuse Large B-cell lymphoma (DLBCL)
Coronary angiography: normal
Magnetic resonance imaging (MRI)/ computed tomography (CT) / FDG- Positron emission tomography (FDG PET): 2 nodules in right ventricle
4
EHJ-CR-D-21-00838

## Slide 5
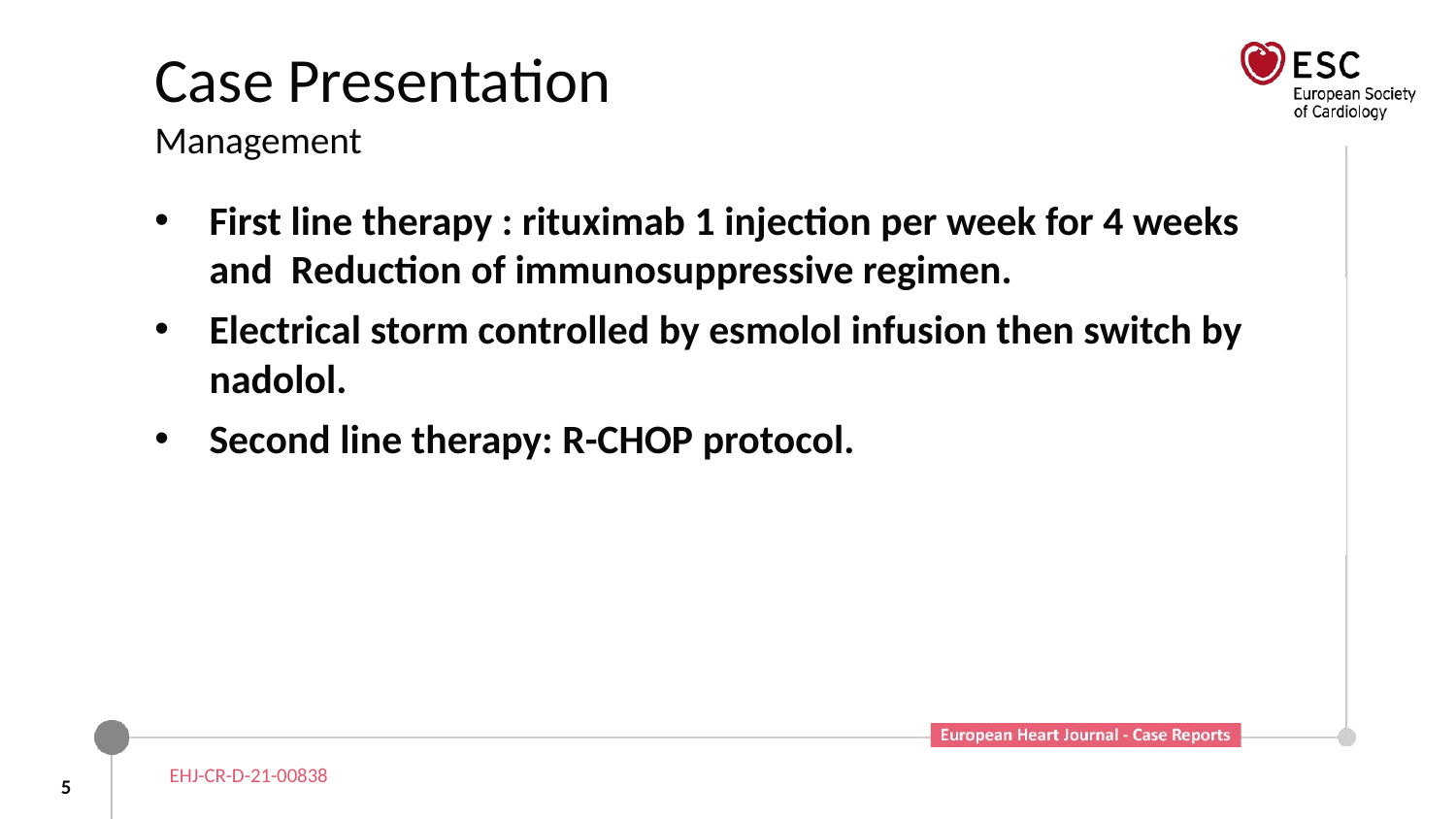

# Case Presentation Management
First line therapy : rituximab 1 injection per week for 4 weeks and Reduction of immunosuppressive regimen.
Electrical storm controlled by esmolol infusion then switch by nadolol.
Second line therapy: R-CHOP protocol.
5
EHJ-CR-D-21-00838

## Slide 6
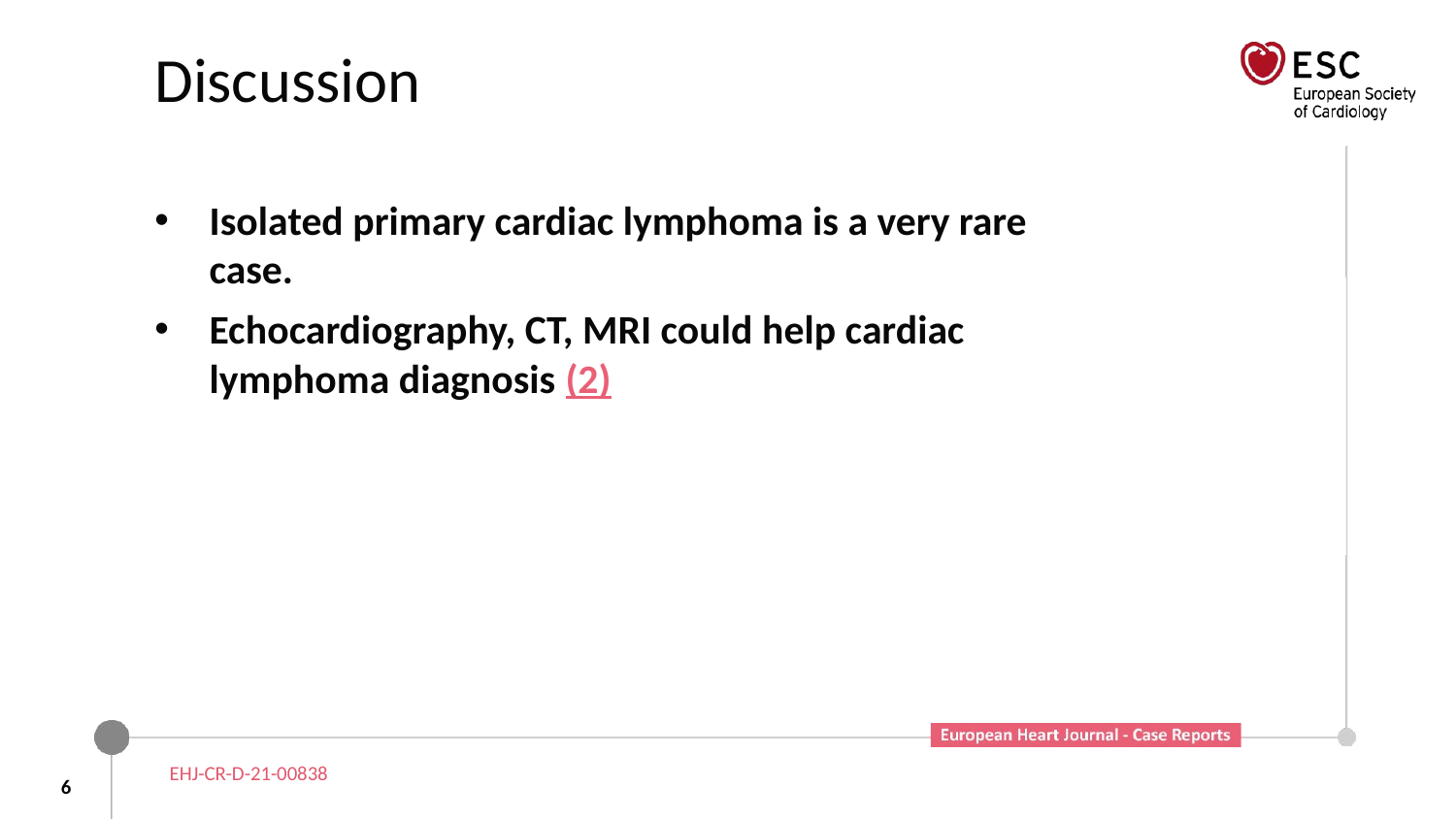

# Discussion
Isolated primary cardiac lymphoma is a very rare case.
Echocardiography, CT, MRI could help cardiac lymphoma diagnosis (2)
EHJ-CR-D-21-00838
6

## Slide 7
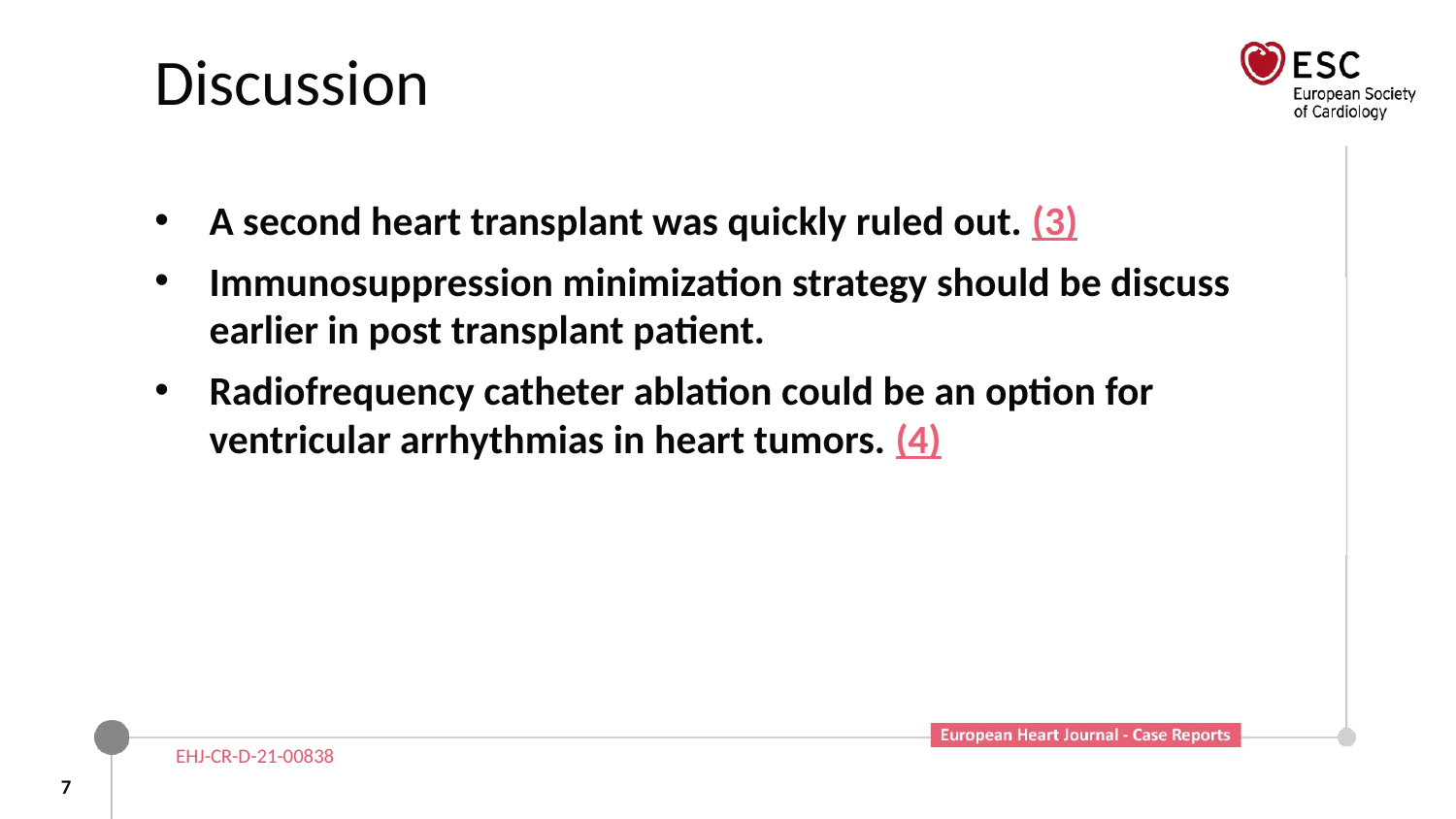

# Discussion
A second heart transplant was quickly ruled out. (3)
Immunosuppression minimization strategy should be discuss earlier in post transplant patient.
Radiofrequency catheter ablation could be an option for ventricular arrhythmias in heart tumors. (4)
EHJ-CR-D-21-00838
7

## Slide 8
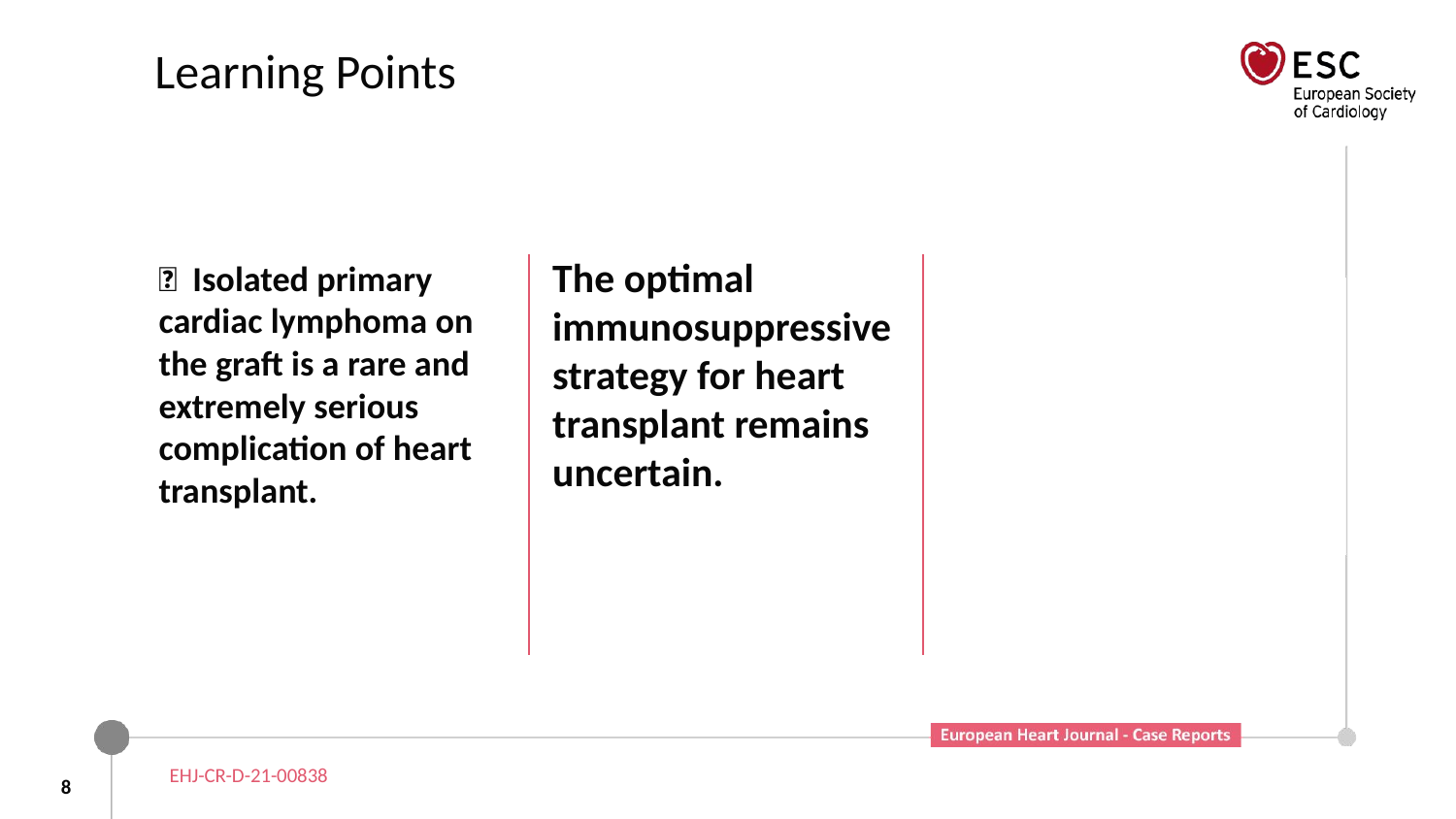

# Learning Points
The optimal immunosuppressive strategy for heart transplant remains uncertain.
  Isolated primary cardiac lymphoma on the graft is a rare and extremely serious complication of heart transplant.
8
EHJ-CR-D-21-00838

## Slide 9
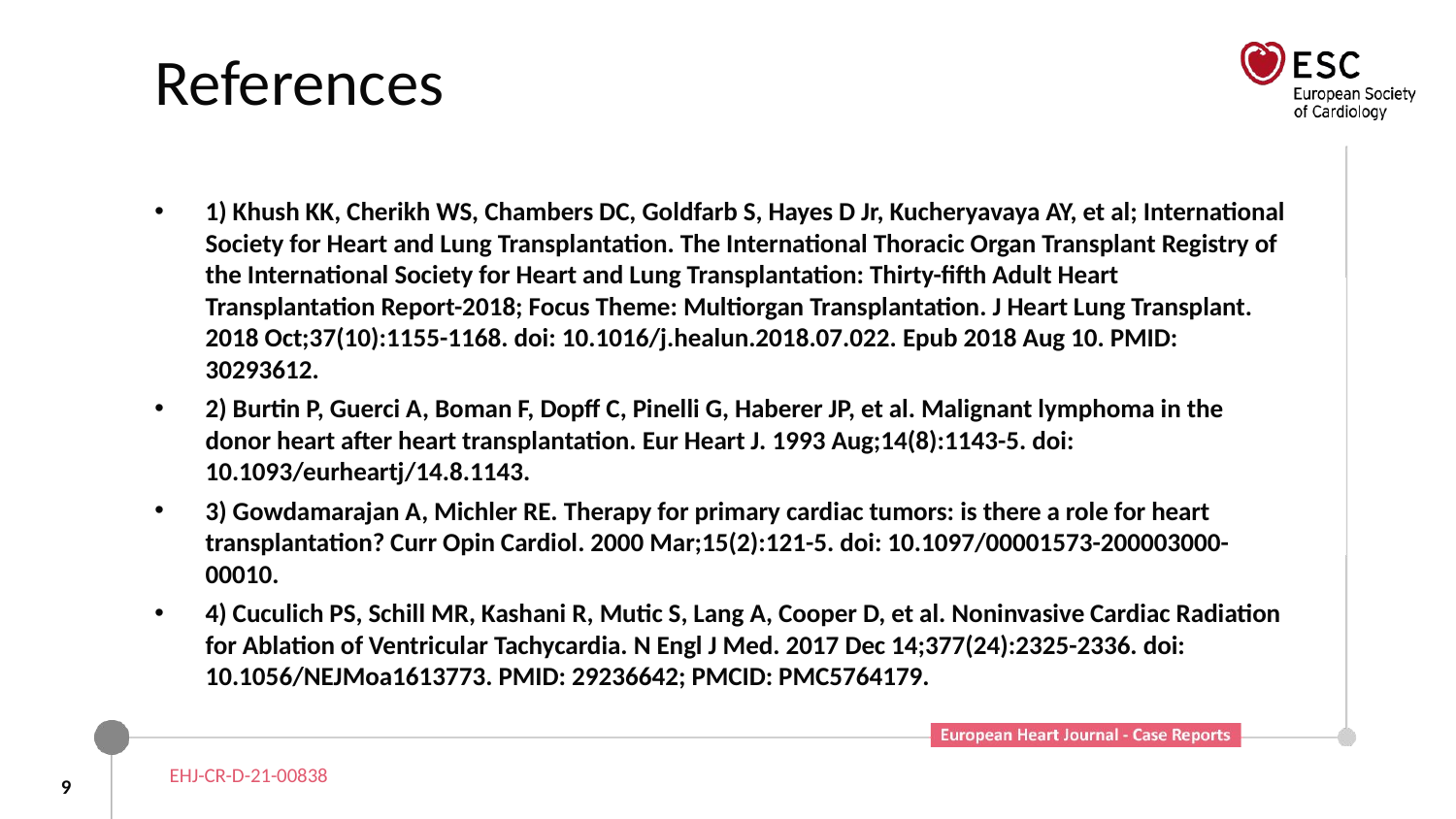

# References
1) Khush KK, Cherikh WS, Chambers DC, Goldfarb S, Hayes D Jr, Kucheryavaya AY, et al; International Society for Heart and Lung Transplantation. The International Thoracic Organ Transplant Registry of the International Society for Heart and Lung Transplantation: Thirty-fifth Adult Heart Transplantation Report-2018; Focus Theme: Multiorgan Transplantation. J Heart Lung Transplant. 2018 Oct;37(10):1155-1168. doi: 10.1016/j.healun.2018.07.022. Epub 2018 Aug 10. PMID: 30293612.
2) Burtin P, Guerci A, Boman F, Dopff C, Pinelli G, Haberer JP, et al. Malignant lymphoma in the donor heart after heart transplantation. Eur Heart J. 1993 Aug;14(8):1143-5. doi: 10.1093/eurheartj/14.8.1143.
3) Gowdamarajan A, Michler RE. Therapy for primary cardiac tumors: is there a role for heart transplantation? Curr Opin Cardiol. 2000 Mar;15(2):121-5. doi: 10.1097/00001573-200003000-00010.
4) Cuculich PS, Schill MR, Kashani R, Mutic S, Lang A, Cooper D, et al. Noninvasive Cardiac Radiation for Ablation of Ventricular Tachycardia. N Engl J Med. 2017 Dec 14;377(24):2325-2336. doi: 10.1056/NEJMoa1613773. PMID: 29236642; PMCID: PMC5764179.
9
EHJ-CR-D-21-00838
